# Adapting clinical guidelines in India—a pragmatic approach

**DOI:** 10.1136/bmj.j5147

**Published:** 2017-11-17

**Authors:** Abha Mehndiratta, Sangeeta Sharma, Nikhil Prakash Gupta, Mari Jeeva Sankar, Francoise Cluzeau

**Affiliations:** 1 Global Health and Development Group, Imperial College London, London W2 1NY, UK; 2 Department of Neuropsychopharmacology, Institute of Human Behaviour and Allied Sciences, New Delhi, India; 3 Quality Improvement Division, National Health Systems Resource Centre, New Delhi, India; 4 Division of Neonatology, All India Institute of Medical Sciences, New Delhi, India

## Abstract

Adapting international guidelines to suit local context can drive evidence based practice in low and middle income countries, say **Abha Mehndiratta and colleagues**, as they describe a pragmatic approach to develop standard treatment guidelines for India

In the past decade, India has witnessed an increase in access to healthcare as it strives for universal health coverage. There has been a rise in publicly financed health insurance initiatives,^1^ and an estimated 185 million people^2^ ^3^ are now covered by some form of health insurance. Although access to healthcare is improving, quality of care remains marred by variations in clinical practice, with problems such as poor diagnosis, irrational use of medicines, and substandard treatment often leading to poor outcomes.^4^ Clear clinical guidance and monitoring mechanisms are urgently needed to improve quality of care, reduce costs, and curtail malpractice.

Clinical guidelines are increasingly used around the world to help change practice and improve patient outcomes by promoting beneficial interventions while discouraging those that are ineffective or possibly dangerous. They assist practitioners in the uptake of credible research into practice^5^ ^6^ by providing recommendations that are informed by a systematic review of evidence.^7^ However, guidelines must also be relevant to the local context.

## Why adapt clinical guidelines

Developing guidelines is complex and resource intensive requiring technical skills and financial support, which are often scarce in low and middle income countries (LMICs). In the absence of evidence based “home grown” clinical guidelines, clinicians resort to recommendations from international guidelines. However, these are not always relevant to their practice because of variations in health systems, resource constraints, and different cultural and social context of patients that drive preferences.^8^

Adapting trustworthy guidelines offers a possible way forward for these countries. Guideline adaptation has been defined as a systematic approach to modify and contextualise evidence based guidelines to suit implementation in the local healthcare system.^9^ ^10^ The process provides an opportunity to systematically consider transferability of recommendations across different settings, including variation in needs, values, costs, and availability of resources.^11^ ^12^ It provides a pragmatic approach for LMICs, especially when relevant and valid guidelines are already available,^13^ ^14^ and helps build local ownership and acceptance, which are essential for guidelines to be adopted.^15^

## Standardised framework for adaptation is needed

Methods and principles for developing high quality guidelines are now well established,^7^ ^16^ ^17^ ^18^ ^19^ but there is no internationally accepted method for adapting them to local contexts.^9^ A recent review identified several frameworks for adapting health related guidelines and indicated a need to evaluate the rigour, efficiency, and transparency of proposed processes.^20^ The lack of international standards can lead to recommendations being adapted on the basis of tradition, anecdote, or low quality evidence, casting doubt on their credibility. This is a major risk, especially for guidelines developed in LMICs, which often score poorly on methodological rigour and editorial independence.^21^ ^22^ ^23^ Improving documentation of the guideline development process and involving methodologists to translate evidence to recommendations can mitigate this risk.^24^ ^25^

## Adapting guidelines for India

In India, clinical guidelines, also called standard treatment guidelines (STGs), are developed at the national and state levels and by a wide range of agencies.^26^ ^27^ ^28^ ^29^ However, the quality of these guidelines is uncertain.^21 ^ In 2014, the Ministry of Health and Family Welfare (MoHFW), convened a guideline task force to develop a road map for standardising the clinical management of diseases in India.^30^ The task force was required to review existing guidelines; recommend principles for review, approval, and regular updating of guidelines; and develop tools for healthcare providers, insurance programmes, medical auditors, and patients to support implementation.

A sample of Indian guidelines reviewed by the task force showed that most guidelines had been adapted from international recommendations but contained little information on the adaptation process, making it difficult to assess their quality. Given time and resource constraints, the task force discounted developing new guidelines and opted to develop, with technical support from its secretariat at the National Health Systems Resource Center, a pragmatic method by which evidence based guidelines could be adapted to suit the Indian context.

In the absence of an internationally accepted adaptation approach, the task force relied on a pilot adaptation framework prepared by the UK National Institute for Health and Care Excellence (NICE) through literature review and expert consensus. A simplified draft guideline development/adaptation handbook was prepared based on the US Institute of Medicine’s principles and standards for a trustworthy guideline.^7^ The aim was to develop guidelines relevant to India using recommendations from existing guidelines whenever possible. New review questions were recommended only for areas not covered by existing guidelines. Fourteen new guideline topics were prioritised and approved by the ministry (table 1).

**Table 1 tbl1:** List of topics for standard treatment guidelines

Clinical specialty	Guideline topic
Critical care and emergency medicine	Snakebite
	Organophosphorus poisoning
Ear, nose, and throat	Acute sinusitis
General medicine	Hypertension—screening, diagnosis, assessment, and management of primary hypertension in adults in India
Mental health	Management of alcohol dependence
	Depression
Neonatology	Detection, management and prevention of hyperbilirubinaemia in term and late preterm newborn infants
	Optimal feeding of low birthweight infants
Obstetrics and gynaecology	Management of recurrent spontaneous abortion
Ophthalmology	Dry eye disease—screening, diagnosis, assessment, and management of dry eye disease in India
Orthopaedics	Management of osteoarthritis knee
Paediatrics	Management of common respiratory infections in children in India
Surgery	The diabetic foot—prevention and management in India
	Major trauma

The adaptation framework used a 10 step approach (fig 1). A multi-stakeholder guideline development group (GDG) was convened for each topic, comprising eminent and experienced professionals working in India in the specified area. They co-opted other members, including patients and patient groups. All GDG members declared conflicts of interest before joining the group.

**Figure f1:**
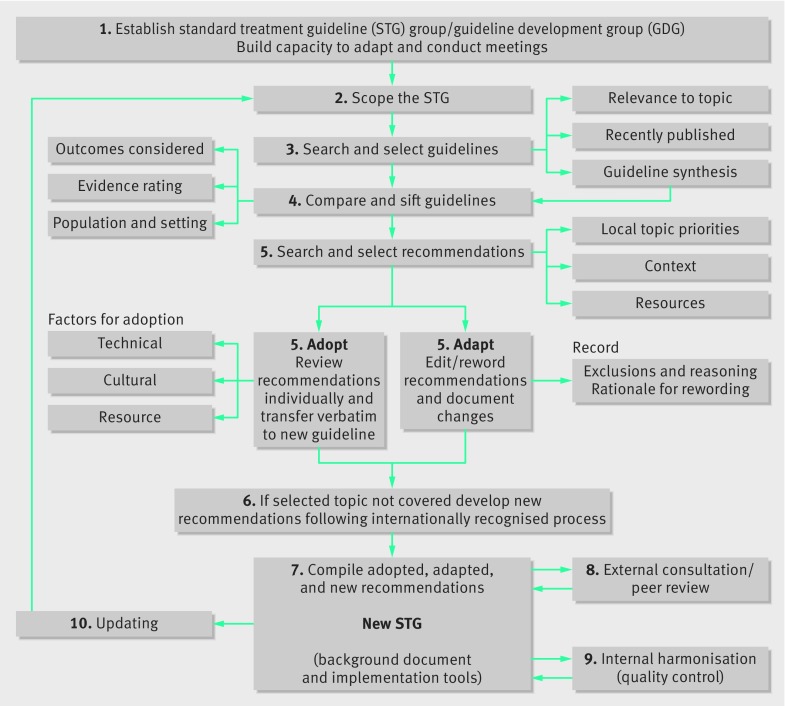
**Fig 1** Ten step adaptation process for developing clinical guidelines in India

A workshop on the guideline adaption methods was held for the GDGs jointly by the National Health Systems Resource Centre and the Global Health and Development Group at Imperial College London (formerly NICE International). The GDG then drafted a scope for the guideline with details of the patient population covered, key clinical issues, relevant healthcare settings, and the main outcomes of interest. The group searched for existing guidelines on the specified topic using the National Guidelines Clearinghouse (NGC), which hosts guidelines that have had a quality check and have been developed, reviewed, or revised within the past five years.^31^ The quality of selected guidelines was not reassessed.

The GDG identified relevant recommendations from these guidelines and decided whether to adopt the recommendation or adapt it for the local context. The reasons for adaptation or for exclusion were documented. These recommendations were compiled to formulate a new comprehensive guideline with clear documentation of the sources for each recommendation. The process took around 8-15 months. A few members of the STG task force and the secretariat plus independent subject experts reviewed the draft guideline, after which it was sent for external peer review, as well as posted in the public domain on the MoHFW website for wider public consultation. Members of the GDG reviewed the feedback and incorporated changes as appropriate. The GDGs were required to provide the task force secretariat with a written explanation of how these comments were dealt with.

The complete guideline, called the “full document” includes detailed methods along with implementation tools such as the quick reference guide for practitioners, a patient information document, and quality standards for measuring compliance to the guideline. The guidelines do not currently include a cost effectiveness analysis.

## Challenges in guideline adaptation

Several lessons emerged from the adaptation process. Although it provided a uniform framework for GDGs, adherence to the steps varied. Documentation of the process of selecting each GDG, the deliberations, and how consensus was reached will help evaluate and refine the methods. Patient involvement was sought and the guidelines were open for public review. However, more robust engagement with patients across different social and cultural groups is required to ensure their concerns and expectations are adequately considered.

Initially, topics such as “approach to acute abdomen” and “joint pains in adults” were selected for guideline development based on the high prevalence and variations in treatment seen in India. However, these were replaced with other topics because it was not possible to find a reliable source guideline on such a broad topic.

The selection of source guidelines was challenging. An internal online survey conducted by the NHSRC among GDG members (28 respondents) showed that 93% of the respondents found some issues relevant to India missing in the source guidelines. However, none of the GDGs conducted a systematic review of evidence on areas where recommendations are lacking because of limited time and expertise.

For some topics, such as snakebite and dry eye, the clearing house did not have all relevant guidelines. For these topics, GDG members identified guidelines through an independent search and assessed the technical quality and the development process using the AGREE II (Appraisal of Guidelines for Research and Evaluation) instrument.^17^ The lack of high quality guidelines was a particular problem for management of snakebite. As urgent guidance was required because of its high prevalence and associated mortality and morbidity, the recommendations were based on a mix of sources, including the World Health Organization guidelines,^32^ observational studies, and clinical expertise of the GDG members. The recommendations were contextualised for practice in remote areas, which are worst affected and lack good health infrastructure.

The GDG judged how far to adapt a recommendation based on members’ expertise, and considering the diverse clinical practice settings in India, resource availability, and affordability. Adaptation ranged from minor edits such as more precise wording to major changes in a clinical recommendation. For example, the GDG on diabetic foot decided that recommending therapeutic footwear proved to relieve plantar pressure during walking was not appropriate because such footwear is not widely available in India. Instead, the GDG made a consensus recommendation on broad footwear features based on members’ collective experience.

In selecting recommendations from multiple guidelines, GDGs found that guideline developers had used different systems for grading the quality of evidence. For example, the same evidence could be graded as II-2, B; C+, 1; or “strong evidence.” This made it difficult to confirm and communicate the strength of a recommendation. To make the recommendations easy to understand and use for providers across the country, the GDGs decided to use words such as “should,” “must,” “do it,” “avoid,” and “don't do it.”

Few GDG members were familiar with evidence based methods, data interpretation, systematic reviews, and synthesis of evidence. This posed problems in determining how far recommendations could be altered without compromising the evidence. To mitigate this risk and enhance transparency, the GDGs were encouraged to document the rationale for adapting recommendations. However, the risk of bias is not completely avoided in the process.

## Moving forward

The pragmatic framework for adapting guidelines marks a step forward in India’s journey towards standardised clinical care and provides a feasible alternative to de novo guideline development for India and possibly other low and middle income countries.

Since the work on STGs in India began, other frameworks for adapting guidelines have been published in the peer reviewed literature, showing the growing interest in this area. One such framework, GRADE-ADOLOPMENT, is an eight step process based on the GRADE working group’s evidence to decision frameworks.^33^ The GRADE-ADOLOPMENT framework emphasises a combination of adoption, adaptation, and, as needed, development of recommendations based on new review questions, similar to the principles put forward in the Indian adaptation process. A global approach on guideline adaptation is needed, building from country experiences to suit different contexts.

The MoFHW has published 12 STGs so far.^26^ Wider dissemination is needed to increase awareness, engender a sense of local ownership and buy-in, and foster use of these recommendations in practice. Identification of a nodal agency for guideline development and implementation is crucial to standardise the process and support updating of guidelines as new evidence and methods become available. The adoption of these guidelines into practice and their effect must be studied. The National Health Policy 2017 recommends the establishment of a national healthcare standards organisation and development of evidence based standard guidelines applicable to both public and private sectors in India.^34^ As India prepares to nearly double its public spending on health by the year 2025,^34^ investment in a robust national programme to produce clinical guidelines will help provide credible guidance on appropriate, evidence based, and ethical practice.

**Key messages**

- Investment is urgently needed in developing clinical guidelines to ensure delivery of quality and ethical healthcare to the Indian population
- As part of a national framework a pragmatic approach was developed to adapt relevant evidence based guidelines to the Indian context commensurate with local resources
- 12 standard treatment guidelines have been published using this method, with explicit documentation of the adoption and adaptation process
- The adaptation framework contributes to wider global efforts to develop a validated approach to producing guidelines relevant to low and middle income countries

## Notes

We acknowledge the contribution of the MoHFW, National Health Systems Resource Centre, the STG Task Force members, all the GDGs, and members of the Global Health and Development Group (formerly NICE International).

Contributors and sources: This article was drafted based on the collective experience of the authors in establishing a national framework for adapting guidelines in India. AM drafted the first version of the manuscript. SS, NG, MS, and FC provided editorial feedback and comments. AM is the guarantor for this article.

Provenance and peer review: Commissioned; externally peer reviewed.

This article is one of a series commissioned by *The BMJ* from the Global Health and Development Group (formerly NICE International) as part of the International Decision Support Initiative (www.idsihealth.org). *The BMJ* retained full editorial control over external peer review, editing, and publication. Open access fees are funded by the Bill & Melinda Gates Foundation.
